# A Factor Analysis of PTSD with Latino Samples with Substance Use Disorders

**DOI:** 10.13188/2330-2178.1000031

**Published:** 2016-08-08

**Authors:** Julia DiGangi, Diana Ohanian, Leonard A. Jason

**Affiliations:** Center for Community Research, DePaul University, Chicago, USA

## Abstract

**Objective:**

The Post - Traumatic Stress Disorder (PTSD) factor structure is not a generally agreed upon concept. It has remained controversial and its’ efficacy regarding different trauma presentations is still in question. Therefore, it is imperative that we evaluate different conceptualizations of the PTSD factor structure. This study aims to understand how PTSD is structured when comorbid with Substance Use Disorder (SUD).

**Method:**

We evaluated presentations of PTSD - SUD from a Latino community based sample. This was done using several accepted models of the PTSD factor structure. We then performed an exploratory factor analysis of the factor structure of PTSD when comorbid with SUD.

**Results:**

We found that the factor structure of PTSD - SUD was different from the structure presented in the DSM - IV and other empirically validated structural models.

**Conclusion:**

The factor structure of PTSD may need to include a separate structure when PTSD is comorbid with SUD. Cultural differences when structuring the PTSD diagnosis should also be considered.

## Introduction

The empirical underpinnings of Post - Traumatic Stress Disorder (PTSD) and its factor structure has been controversial [1–4]. The DSM - IV - TR utilized three central domains of dysfunction: symptoms related to re-experiencing the traumatic event (Cluster B), persistent avoidance of stimuli associated with the traumatic event (Cluster C), and persistent symptoms of increased arousal (Cluster D) [5]. However, it was expert consensus, rather than data that influenced the DSM - IV’s symptom clusters [6]. For almost two decades there has been a lack of clarity regarding the symptom structure; findings from a range of studies supported two - [2,7,8], three - (e.g. DSM-IV), four - [4,9] and five - factor (e.g. Pietrzak RH et al. [10]) models of PTSD with DSM-IV.

The lack of reliability and validity surrounding DSM - IV’s version of PTSD has, in part, necessitated new diagnostic criteria for the disorder in DSM - 5. DSM - 5 divided the three clusters of PTSD into four clusters [11]. Cluster C (avoidance/numbing) was separated into two clusters: Cluster C (avoidance) and Cluster D (negative alterations in cognitions and mood). In addition, the DSM - IV - TR criterion of having experienced helplessness or horror as a result of the trauma was removed in DSM - 5. The DSM - 5 also has added symptoms to the diagnosis, creating a veritable “menu” to, as Friedman of the DSM’s revision committee said, “cover all presentations of [of PTSD]” [12]. As the DSM - 5’s structure is still in its’ infancy and not yet empirically validated many researchers are still using the DSM - IV. Moreover, the DSM - 5’s criterion is not in line with other popular empirically validated structural models. Therefore, we decided to investigate the applicability of the most popular models in this study.

We chose two highly validated models to test. King et al. has developed one of the most empirically supported and popular symptom structure models for PTSD [8]. Using a sample of 524 male military veterans, King and colleagues identified four factors, which included: 1) Re-experiencing, 2) Avoidance, 3) Numbing, and 4) Arousal. Another empirically supported model was proposed by Simms et al. who used a sample of veterans from the first Gulf war in 1991 [4]. Simms et al.’s model was based on a theoretical assumption that PTSD includes a dimension of general distress or negative affectivity, which can be seen in all anxiety and depressive disorders [4]. The four factors include: 1) the general distress factor, which they named the dysphoria factor, 2) an avoidance factor, 3) a hyperarousal factor, and 4) a re-experiencing symptoms factor. A recent meta-analysis of PTSD’s symptom structure, which included 14,827 participants across studies, found that it was Simms et al.’s model - as opposed to King’s - that yielded the best fit across studies.

Finding a suitable model for PTSD is challenging due to the unique nature of the disorder. PTSD is a complex psychiatric disorder in that it is not agnostic to etiology. Unlike other disorders, it requires a certain type of event to precede diagnosis. In order to cope with the traumatic event and the subsequent distressing symptoms, individuals with PTSD often utilize alcohol and drugs. Consequently, PTSD and substance abuse disorders (SUD) are highly comorbid [13–20]. According to Pietrzak RH and colleagues, 46.4% of people with PTSD have substance abuse disorder [10]. Moreover, for men with PTSD, alcohol abuse and dependence is the most common co-occurring disorder [17,18]. Similarly, studies of women with PTSD have demonstrated that alcohol abuse and dependence are highly comorbid, with only depression and other anxiety disorders being more common [17,18].

This level of comorbidity and lack of clarity regarding treating PTSD when it is comorbid with SUD underlies the necessity of more research into the complexity of PTSD - SUD. The high comorbidity between PTSD and SUD indicates that the disorders are functionally related [21]. However, we still do not understand the exact manner in which PTSD and SUD are related. Some believe that substance abuse increases the risk of being exposed to trauma while other believes that substance use function as “self-medication”. Still others (Schäfer and Najavits) have pointed to genetics as the reason for the relationship between PTSD and SUD [22,23]. Preliminary work into the factor structure of PTSD - SUD has suggested an entirely different factor structure for PTSD - SUD than PTSD alone. Scoboria A et al. examined the factor structure of PTSD features with a sample of adult trauma survivors in substance abuse treatment and found a factor structure very different from that which is typically utilized when conceptualizing PTSD absent comorbid SUD [24]. In their analysis, they found five factors representing demoralization, somatic dysregulation, anger dysregulation, risk for self-harm and altered sexuality. Bonin et al. suggest that in order to treat either one of these disorders one must understand how their presentation is fundamentally different when paired [25]. In their study, they confirmed the highly comorbid nature of these diagnoses; 52.8% of participants from a substance abuse rehab clinic were diagnosed with PTSD. It is still unclear how best to understand the potential heterogeneity of PTSD symptomology in the context of SUD.

Another factor that contributes to the complexity of PTSD is the issue of culture. Cross-cultural research has indicated the factor structure of PTSD varies considerably depending on context [26,27]. Further highlighting the cultural variability expressed in PTSD, various studies have demonstrated conflicting evidence as it relates to how PTSD impacts Latinos. Some studies have found that Latinos are at greater risk than Black and White counterparts for PTSD diagnosis and other research has suggested that Latinos are at equal or lesser risk [17,28,29]. The lack of research on Latinos is particularly problematic given that Latinos represent the largest and fastest -growing minority group in the United States. According to the 2010 U.S. census, an estimated 50.5 million Latinos live in the U.S. [30]. Current census trends suggest the population will continue to increase, reaching 102.6 million by 2050 and constituting nearly a quarter of the country’s population.

The present study examined PTSD in a community - based sample of Latinos who were in recent recovery from SUDs. The factor structure of PTSD was explored through an exploratory factor analysis in order to determine what patterns of symptoms appeared among this group.

## Methods

### Study sample and design

This study was part of a NIH funded study that examined Latinos use of substance abuse aftercare, called Oxford Houses [31]. Oxford Houses are self-run recovery homes in which same-sexed individuals live together to establish sobriety, democratically run living environment; they are the largest residential self-help recovery program in the U.S [32]. After IRB approval was obtained, participants for this study were recruited from multiple community-based organizations and health facilities from a large metropolitan area in the Midwest. Inclusion criteria required that participants 1) were Latino and 2) had completed a substance abuse treatment program. At the time of this study, all participants had completed substance use treatment and, therefore, no one was using substances. Interviews were completed by bilingual research assistants and participants received $30 after completing the interview. This sample consisted of 104 Latinos. See Table 1 for more details.

### Measures

Section E of the Diagnostic Interview Schedule-IV, which is based upon the Diagnostic and Statistical Manual of Mental Disorders, Fourth Edition (DSM - IV) was used to assess participants’ PTSD symptoms as well as the nature and number of Criterion A events experienced [6,33]. Participants who endorsed experiencing multiple traumatic events, were asked to nominate a WTE (Worst Traumatic Event) and to say at what age this event occurred. Examples of WTEs included sexual assaults, incest, physical abuse, and sudden death of friend/family member. The DIS was used because nonclinicians (i.e. research assistants) are able to administer this measure to participants. The PTSD section of the current version of the DIS was tested for reliability and validity in a study of substance abusers and had fair to good inter-rater reliability (kappa 0.40 – 0.67). The results are consistent with the literature on reliability of PTSD with substance abusers. While an English version of the DIS - IV was available, there was no published or commonly used Spanish version. A team of translators worked to translate and back-translate the DIS. The translators were native Spanish speakers from Spanish-speaking locales that were appropriate for the study’s participants in Chicago (e.g. Mexico, Puerto Rico). Furthermore, the Spanish version was piloted on Latinos with SUDs and was implemented clearly and effectively.

## Results

An exploratory factor analysis was then undertaken in MPlus. The goal of EFA is to distill a large number of observed variables (i.e. items) into a smaller number of factors [34]. The estimating technique that used and the extraction technique that was used was weighted least squares and variance adjusted (WLSMV). WLSMV was used as the estimating technique because of the non-normality of the distribution of data (i.e. data were a binomial distribution due to the yes/no scoring of the DIS). Geomin rotation, the default rotation for the MPlus software package, was used. Based on theory, five various EFA models were tested with MPlus (i.e. a 1 - factor; 2 - factor; 3 - factor; 4 - factor; and 5 - factor). Models results are presented in Table 2.

All models were evaluated through the following goodness - of - fit indices: SRMR, CFI and RMSEA. According to Hu and Bentler good model fit was indicated with values of SRMR < 0.08, CFI > 0.95, and RMSEA < 0.05 [35]. Next, the goodness-of-fit indices were examined (Table 2). Given Hu and Bentler’s cutoffs for SRMR, CFI, and RMSEA delineated above, the one and two factor model were excluded [35]. The five factor model failed to converge, and models with more factors were not examined because they had no meaningful theoretical basis.

In the examination of the three and four factor model, the four-factor model, comprised of factors named Approach/Avoidance, Fear, Hyperarousal and Numbing, made most conceptual sense in that it is most consistent with prior empirical investigation as well as the theoretical underpinnings of PTSD (See Table 3 for loadings of four factor model).

## Discussion

Results from the EFA indicated that a four-factor solution was the best fit of the data. Factors were named Approach/Avoidance, Fear, Hyperarousal and Numbing. In terms of the Approach/Avoidance factor, one of the hallmark features of PTSD is a feedback loop that vacillates between recurrent, intrusive memories of the trauma followed by a subsequent avoidance of trauma-related cues. In fact, the approach - retreat cycle is a key mechanism theorized to sustain PTSD [36,37]. Trauma survivors often re-experience the traumatic event(s) in the forms of recurrent thoughts, flashbacks and emotional distress at reminders of the trauma. Given the nature of traumatic memory, these memories can be disjointed, chaotic and incomplete accounts of the traumatic event. When these distressing memories intrude into conscious awareness, the coping response is often to blunt - or avoid - the processing. Avoidance is a critical defence mechanism, protecting individuals from the distress reminders of the trauma evoke.

This oscillation process between approach and avoidance has analogues that occur on multiple levels and across multiple points in time. For instance, the approach-avoidance dyad can happen in a moment: an individual may spontaneously begin to experience sensory reminders of the trauma (e.g. a smell), and then immediately squelch further processing. It can also pervade key domains of functioning for substantial periods of time. Individuals may drastically alter major component of their life (e.g. jobs, locations, social networks), if they believe these domains to represent intrusive reminders of the trauma. For example, it is not uncommon for someone suffering from PTSD after a car accident to avoid car travel in its entirety. Finally, this approach-avoidance cycle also appears to have a neurobiological basis. Just as the approach-avoidance cycle is playing out on a psychological level, it also has a neurobiological analogue. Specifically, the amygdala, insula, ventral striatal, and prefrontal regions appear to be neural substrates that underpin the approach-avoidance cycle [38]. In sum, research from various domains highlight that the approach-avoidance is a key aspect of PTSD’s psychopathophysiology. Thus, the joining of these two phenomena under one factor is consistent with consistent with theory laid out by many PTSD researchers [36,37]. In fact, Taylor, Kuch, Koch, Crockett, and Passey conducted a factor analysis of PTSD and concluded that PTSD was comprised of two factors - one factor was called Hyperarousal/Numbing and the other was called Intrusions/Avoidance [7]. Although this factor model has not received empirical support, it draws on the same theories of Horowtiz and Foa EB et al. to provide a theoretical rationale that joined these constructs into a single factor [36,37].

In the present study, eight of 17 items loaded on the Approach/Avoidance factor. Three of these eight items relate to approach-related phenomena in that describe processes by which the trauma enters conscious awareness. These three items include: “Did being reminded of the trauma or being in a similar situation make you feel very upset or anxious?;” “Did you ever suddenly feel that you were experiencing the trauma all over again?;” “After the trauma, have you kept thinking about it again and again when you did not want to?” The remaining five items relate to avoidance behaviours: “After the trauma, did you try to avoid thinking or talking about it?;” “Did you stay away from certain places, people, or activities to avoid being reminded of it?;” “After the trauma, did you lose interest in activities that were once important or enjoyable to you?;” “ Did you start to feel more isolated or distant from other people?;” “Have you found it more difficult to have love or affection for other people?”

The grouping of these eight items under a single Approach/Avoidance factor represents a diversion from both the four factor model put forth by the DSM - 5, modeled off of Simms et al. as well as the three factor model of the DSM-IV [4]. Although both the DSMIV and the DSM-5 have factors that describe approach and avoidance related behaviours, these two constructs appear on two separate factors. In this sample of Latinos in PTSD-SUD, the approach and avoidance related behaviours loaded onto a single factor, and - as noted, there is theoretical precedent for the co-existence of approach and avoidance symptoms into a single factor.

The second factor, named the Fear factor, described items that are related to fear. At its core, PTSD is a fear-based disorder that is predicated on the experience of fear, horror, and/or helplessness [5]. Although the experience of fear is nearly universal during a trauma, the pathogenesis of PTSD is predicated on an unremitting fear response [39]. In the present study, there were four items that loaded on to this factor. These included: “Afterward the trauma, did your concern about danger increase, and did you become much more careful than before?;” “Did you become jumpy or get easily startled by ordinary noises or movements?;” “Did you keep having bad dreams or nightmares about it?;” “After the trauma was over, were you having more trouble than usual falling asleep or staying asleep?”

As a fear response is integral component of PTSD’s etiology, an argument could be made that, on some level, each of the 17 symptom questions is predicated on fear. However, these four items most directly describe a fear response. For example, increased concern about safety and feeling jumpy are typically direct correlates of fear. Moreover, the symptoms of PTSD - as detailed earlier - encompass exceptional heterogeneity [40]. While fear is indisputably a fundamental component of PTSD, some of the 17 symptoms that embody PTSD also describe feelings of irritability, anger and depression. The four items that loaded on the Fear factor were conceptualized as more direct byproducts of fear response whereas other items are more ambiguous. For example, Item 7 asks, “Did you realize that your heart would pound, you would sweat, or you become physically sick when you were reminded of the trauma?” While fear could certainly motivate such a response so, too, could anger.

The Hyperarousal factor in this study was comprised of three items. These included: physiological reactivity to trauma cues, irritability and difficulty concentrating. The Hyperarousal factor confirmed what had long been recognized as a core feature of PTSD. Since the inception of PTSD into the DSM - III, psychophysiological arousal has been a hallmark feature of PTSD’s pathology - and while there continues to be considerable debate about the precise factor structure of PTSD, one of the least ambiguous aspects is that arousal is indeed a pathognomonic marker of the disorder [41]. Strong autonomic arousal, fear response, and fight - flight tendencies are core to the psychopathophysiology of certain PTSD symptoms [42]. Furthermore, many factor analytic studies have supported the notion that hyper-responsiveness to fear is a core component of PTSD’s pathology [1,4,5,8,43]. Although there is virtually no debate as to whether hyperarousal is a component of PTSD etiology, there are discrepancies as to which PTSD symptom items best represent this construct. For example, in Simms et al. only two items related to hypervigilance and exaggerated startle response whereas in the King et al. model five items comprised the hyperarousal factor: sleep disturbance, irritability, difficulty concentrating, hypervigilance, and exaggerated startle response [4,8].

The Numbing factor in this study was comprised of two items. These included a sense of a foreshortened future and an inability to recall aspects of the trauma. This factor is consistent with earlier work that has routinely identified emotional numbing as a core component of the disorder [5,8]. Emotional numbing, as defined by the DSM - IV, is blunting and a lack of general responsiveness that was not present before the trauma. The DSM - IV grouped numbing-related behaviours with avoidance-related behaviours under a single factor, called Avoidance/Numbing. Subsequent research exploring the psychological and neurobiological underpinnings of avoidance and numbing have suggested that they are likely two distinct constructs. For example, Foa EB et al. have suggested that different mechanisms drive avoidance and numbing behaviours [36]. Avoidance, they argued, requires volitional processes that actively abort the intrusive (i.e. approach) symptoms of PTSD whereas numbing is an automatic and unconscious process that involves emotional blunting. Using neuroscience as a mechanism to explain the basis of numbing behaviours, Foa EB and colleagues suggested that numbing may result from overstimulation of the endogenous opioid system, which causes an analgesic effect [36]. Several other scholars have adopted this view [2,7]. Along the same line, Kolb introduced a neuropsychological explanation of PTSD, suggesting that emotional blunting may be caused by changes at the neuronal level, while avoidance may be an attempt to curb the physiological arousal that typically accompanies intrusive memories of the trauma [44]. This was separated in the DSM - 5 into avoidance and negative alterations in cognitions and mood.

Further evidence that the constructs of numbing and avoidance are not tapping the same latent phenomena comes from factor analytic studies that have shown numbing and avoidance to represent separate factors [1,8,45]. Although there is clear evidence that avoidance and numbing should be separate factors, there is little debate as to whether numbing is a part of PTSD’s symptom constellation. Instead, the debate arises around the items that load on these factors. For example, the DSM - IV currently has seven items (i.e. avoiding thoughts, avoiding reminders, inability to recall aspects of trauma, loss of interest, detachment, restricted affect, sense of foreshortened future) that corresponded to the numbing cluster whereas King et al. found five items (i.e. inability to recall aspects of trauma, loss of interest, detachment, restricted affect, sense of foreshortened future) [8]. Another study by Amdur and Liberzon, that used the 15 - item Impact of Events Scale (IES) found three items that loaded on the numbing factor (i.e. items dealing with feelings of numbness and that the event was surreal) [46]. Thus, while many studies acknowledge that numbing is a core component of PTSD’s pathology, there is debate around what precise items comprise this factor.

As mentioned, results from the current study indicated that items related to amnesia and hopelessness about the future load onto the numbing scale. Breslau N and colleagues indicated that psychogenic amnesia was rarely endorsed [47]. The rarity and severity of these items on this factor may make sense in the context of this sample of individuals who have exceptionally complex substance abuse and trauma histories.

This study has several limitations. Recall bias exists in any study that requires participants to reflect on past events, nowhere is recall bias more profound than in the recall of traumatic memories. Ample research has demonstrated that current psychological symptoms can alter memories of behaviours and experiences prior to, during and after a trauma [48–50]. Complicating the issue further, the presentation of PTSD symptoms in individuals with comorbid PTSD - SUD is more severe than individuals suffering from PTSD alone [51,52]. Thus, if symptomatology affects recall, and comorbid PTSD - SUD leads to greater symptomatology, then the problem of recall bias was potentially a substantial issue in this sample of individuals in recovery from substance abuse. Future studies that prospectively assess the relationship between trauma and substance abuse would represent a valuable contribution to elucidating the etiology of comorbid PTSD and SUDs. A second limitation has to do with the measurement of trauma and, subsequently, PTSD in this sample of multiply traumatized individuals with SUDs. To assess PTSD, this study used the Diagnostic Interview Schedule (DIS), a highly respected instrument, with good psychometric properties that is a direct analogue to the DSM-IV’s criteria for PTSD. For example, the 17 symptoms that comprise PTSD are directly converted into questions in the DIS. An additional benefit of the DIS is that it can be administered by non-clinicians, thereby increasing its usability. Thus, as the DIS is a direct correlate to the PTSD diagnosis, the problems with the DIS elucidate not only problems with the instrumentation, but with the diagnostic criteria as a whole. The fourth limitation has to do with the sample. First, women were under represented in this study. The sites from which we recruited were populated by very few females. Furthermore the sample size was quite small. Therefore, it is difficult for us to generalize our findings. Moreover, although many studies are beset by problems associated with sample size, the sample size issues associated with this study speak to a host of larger issues relating to accessibility of treatment.

In sum, this exploratory factor analysis, conducted on a sample of 104 Latinos in RSUD indicated that a four factor model was the best fit of the data. These factors included Approach/Avoidance, Fear, Hyperarousal, and Numbing. Contrary to the DSM - 5’s model we found that Approach/Avoidance work together. Furthermore, we found that fewer symptoms loaded on to Fear and that other more ambiguous items could be thought of as anger rather than fear. Hyperarousal was confirmed as a core feature of PTSD and remains the least ambiguous feature in the structural conceptualization of PTSD. Finally, Numbing was found to be integral, rather than part of an offshoot of another factor (as in the DSM - 5) for the comorbid structure of PTSD-SUD. Numbing included symptoms of amnesia which demonstrates the severity, uniqueness, and therefore necessary reconceptualization of PTSD - SUD.

Despite high comorbidity between PTSD and SUD, current guidelines for PTSD treatment do not explain whether one should alter treatment for PTSD when it is comorbid with SUD [53]. Our findings regarding the unique factor structure of PTSD-SUD demonstrate that this lack of clarity regarding treatment of PTSD-SUD might be detrimental to the treatment of persons struggling with both of these disorders. After reviewing many studies that treated persons with PTSD-SUD, Roberts et al. found that treatments that addressed both PTSD and SUD were more successful than treating PTSD alone [54]. However, the treatment effects were small. This small effect could be due to the high dropout rate found in most of these studies, which is often a problem in PTSD treatment. Therefore, we suggest a treatment approach that would target the aforementioned factors and attempt to address tolerability issues in PTSD treatment. As we demonstrated, one of the most important elements to keep in mind when making treatment more tolerable might be attention to cultural differences.

## Figures and Tables

**Table 1:** Demographic data

n (%)		Total (n = 104) n (%)	Puerto Rican (n = 47) n (%)	Mexican (n = 39) n (%)	Other (n = 18) n (%)	*p*
** Sex**	Male	87 (83.7)	40 (85.1)	32 (82.1)	15 (83.3)	-
	Female	17 (16.3)	7 (14.9)	7 (17.9)	3 (16.7)	-
**Immigration status**	Natural born citizen	75 (72.1)	39 (83.0)	25 (64.1)	11 (61.1)	-
	Immigrated	29 (27.9)	8 (17.0)	14 (35.9)	7 (38.9)	-
**Marital status**	Never married	57 (54.8)	22 (46.8)	22 (56.4)	13 (72.2)	-
	Divorced	22 (21.2)	12 (25.5)	8 (20.5)	2 (11.1)	-
	Separated	20 (19.2)	11 (23.4)	8 (20.5)	1 (5.6)	-
	Married	4 (3.8)	1 (2.1)	1 (2.6)	2 (11.1)	-
	Remarried	1 (1.0)	1 (2.1)	0 (0.0)	0 (0.0)	-
**Primary substance of choice**	Alcohol & other drug	28 (26.9)	11 (23.4)	10 (25.6)	7 (38.9)	-
	Alcohol	24 (23.1)	9 (19.1)	12 (30.8)	3 (16.7)	-
	Heroin	24 (23.1)	14 (29.8)	8 (20.5)	2 (11.1)	-
	Cocaine	13 (12.5)	7 (14.9)	2 (5.1)	4 (22.2)	-
	Cannabis	11 (10.6)	4 (8.5)	5 (12.8)	2 (11.1)	-
	Two or more, not alcohol	3 (2.9)	2 (4.3)	1 (2.6)	0 (0.0)	-
	Other opiates/analgesics	1 (1.0)	0 (0.0)	1 (2.6)	0 (0.0)	-
**Severity of use**	Experienced alcohol DT	15 (14.4)	5 (11.1)	8 (20.5)	2 (11.8)	-
	Experienced a drug OD	40 (38.5)	21 (44.7)	16 (41.0)	3 (16.7)	-
	Prior alcohol abuse treatment	55 (53.4)	17 (36.2)	24 (63.2)	14 (77.8)	0.003
	Prior drug abuse Treatment	79 (76.0)	39 (83.0)	27 (69.2)	13 (72.2)	-
**Substance abuse treatment**	Inpatient	75 (72.9)	35 (74.5)	30 (76.9)	10 (55.6)	-
	Recovery home	9 (8.7)	2 (4.3)	3 (7.7)	4 (22.2)	-
	Outpatient	6 (5.8)	4 (8.5)	2 (5.1)	0 (0.0)	-
	Transitional program	3 (2.9)	0 (0.0)	1 (2.6)	2 (11.1)	-
	Department of corrections	3 (2.9)	0 (0.0)	1 (2.6)	2 (11.1)	-
	AA/NA	2 (1.9)	1 (2.1)	1 (2.6)	0 (0.0)	-
	Shelter	2 (1.9)	1 (2.1)	1 (2.6)	0 (0.0)	-
	24 hour AA sites	2 (1.9)	2 (4.3)	0 (0.0)	0 (0.0)	-
	Detox	1 (1.0)	1 (2.1)	0 (0.0)	0 (0.0)	-
	No treatment	1 (1.0)	1 (2.1)	0 (0.0)	0 (0.0)	-
**PTSD**	Lifetime PTSD	48 (46.2)	21 (44.7)	19 (48.7)	8 (44.4)	-
	Current PTSD	28 (26.9)	14 (29.8)	9 (23.1)	5 (27.8)	-
		**M (SD)**	**M (SD)**	**M (SD)**	**M (SD)**	***p***
**Age**		37.1 (10.5)	39.6 (9.1)	33.5 (10.2)	38.5 (12.9)	0.021
**Years of Education**		11.5 (2.0)	11.7 (2.1)	11.0 (2.0)	11.6 (1.9)	-
**Years Incarcerated**		3.5 (5.7)	4.0 (6.2)	1.8 (3.5)	5.5 (7.5)	-
**Severity of use**	Alcohol DT a	9.2 (7.8)	7.2 (7.4)	8.4 (6.3)		-
	Drug OD a	3.0 (3.9)	3.7 (4.9)	2.4 (2.7)	2.0 (1.0)	-
	Prior alcohol abuse treatment a	3.2 (3.9)	3.9 (5.7)	3.0 (2.9)	2.6 (2.6)	-
	Prior drug abuse treatment a	5.5 (8.3)	6.1 (6.7)	5.9 (11.6)	2.9 (2.7)	-
	Alcohol DT b	1.4 (4.4)	0.8 (3.2)	1.7 (4.4)	2.1 (6.7)	-
	Drug OD b	1.2 (2.8)	1.7 (3.7)	1.0 (2.0)	0.3 (0.7)	-
	Prior alcohol abuse treatment b	1.7 (3.2)	1.4 (3.9)	1.9 (2.7)	2.1 (2.5)	-
	Prior drug abuse treatment b	4.2 (7.6)	5.1 (6.6)	4.1 (10.0)	2.1 (2.6)	-
**Trauma**	Criterion A events	6.4 (2.7)	6.8 (2.5)	5.8 (2.7)	6.6 (3.0)	-

a Mean of participants who experienced given event (i.e. alcohol DT, drug OD, prior treatment)

b Mean of entire sample, including those who did not experience given event.

**Table 2:** EFA fit statistics

	X^2^	*df*	CFI	SRMR	RMSEA
1-Factor Solution	51.01	35	0.963	0.118	0.067
2-Factor Solution	32.95	33	1.000	0.085	0.000
3-Factor Solution	23.91	34	1.000	0.068	0.000
4-Factor Solution	20.30	30	1.000	0.058	0.000

**Table 3:** Factor loading for section E of the Diagnostic Interview Schedule – IV

Section Items	DIS factors			
	Factor 1	Factor 2	Factor 3	Factor 4
Unwelcome recurring thoughts	0.50	-	-	-
Sudden re-experiencing	0.74	-	-	-
Upset/anxiety brought on by similar situation	0.80	-	-	-
Discussion/thought avoidance	0.58	-	-	-
Stimuli avoidance	0.62	-	-	-
Loss of interest	0.98	-	-	-
Feeling isolated	0.97	-	-	-
Difficulty with affection	0.78	-	-	-
Increased caution/concern with danger	-	0.39	-	-
Easily Startled	-	0.99	-	-
Nightmares concerning trauma	-	0.54	-	-
Difficulty Sleeping	-	0.60	-	-
Physical arousal/discomfort	-	-	0.35	-
Increased irritability	-	-	0.73	-
Trouble focusing	-	-	0.68	-
Hopeless attitude towards the future	-	-	-	0.55
Inability to recall aspects of trauma	-	-	-	0.64
**Cronbach’s α**	**0.83**	**0.65**	**0.70**	**0.41**
